# In a Changing World—An Economical Comparison Between Traditional and Wet-And-Drought-Resistant Grasses in Swedish Cattle Production Under Different Weather Scenarios

**DOI:** 10.3390/ani15030295

**Published:** 2025-01-21

**Authors:** Kristina Holmström, Karl-Ivar Kumm, Hans Andersson, Mikaela Jardstedt, Dannylo Sousa, Anna Hessle

**Affiliations:** 1 Department of Applied Animal Science and Welfare, Swedish University of Agricultural Sciences, P.O. Box 234, 53223 Skara, Sweden; karl-ivar.kumm@slu.se (K.-I.K.); mikaela.jardstedt@slu.se (M.J.); dannylo.sousa@slu.se (D.S.); anna.hessle@slu.se (A.H.); 2 Research and Development, Rural Economy and Agricultural Society Sjuhärad, P.O. Box 5007, 51405 Länghem, Sweden; 3 Department of Economics, Swedish University of Agricultural Sciences, P.O. Box 7013, 75007 Uppsala, Sweden; hans.andersson@slu.se

**Keywords:** profitability, climate change, dairy cow, bull, beef cow

## Abstract

This study compared the profitability when feeding silages of different grass species in enterprises with either dairy cows, beef breed bulls, or beef suckler cows. Traditional (TR) grasses timothy and meadow fescue was compared to the alternative wet-and-drought-resistant (WD) grasses tall fescue, festulolium, and reed canary grass in three different weather scenarios with either normal conditions (Ref), delayed late harvest time due to wet weather conditions (Wet), or decreased grass yield due to dry weather conditions (Dry). Contribution margin calculation was conducted for three geographical regions in Sweden. In the Ref and Wet scenarios, TR was more competitive than WD for dairy cows and beef bulls in all regions. Also in the Dry scenario, TR was more competitive than WD for dairy cows, as the lower production cost of the WD was outweighed by a lower milk yield of cows fed WD compared to cows fed TR. Contrary, for beef bulls, WD gave a higher contribution margin than TR did in the Dry scenario, where the break-even for WD being superior over TR occurred when more than every second year was dry. WD reed canary grass was always more competitive than TR and WD festulolium for beef cows.

## Simple Summary

The aim of this study was to compare profitability when feeding silages of different grass species to investigate if animal production or cost of silage is most important for profitability in production with cattle. Contribution margin calculations was conducted for three different geographical regions in Sweden for enterprises with either dairy cows, beef breed bulls, or beef suckler cows. Calculations were conducted for three different weather scenarios with either normal, delayed harvest due to wet weather conditions, or drought conditions. Even if there was less yield of grass of timothy, it was superior to tall fescue fed to dairy cows. For beef breed bulls, it depended on weather conditions if meadow fescue or tall fescue was the best choice. Reed canary grass fed to pregnant beef cows was always superior to festulolium and a mix of meadow fescue—timothy. The result from this study is of importance to farmers’ decision-making in a changing climate.

## 1. Introduction

Forecasted climate changes will steadily amplify challenges in crop cultivation and thereby also animal production. There is hence a need for fodder crops to be more resilient to extreme weather conditions, which in northern Europe means drought, heat, flooding, and reduced snow cover in winter [1,2]. During the last fifty years, the climate changes in Sweden have led to a higher average temperature and annual precipitation, a shorter winter but longer vegetation period, and an increased number of hot days. The frequency and magnitude of long-term droughts, storms, and heavy rainfalls have increased. These changes will continue but with variation among geographical regions. The largest increases in temperature and precipitation are forecasted to occur during winter and in northern Sweden, whereas the increase in precipitation during winter will be the least in the southeastern part of the country [2]. Additionally, the number of days with no precipitation at all during summer will increase in southern Sweden. Taken together, these climate changes affect the crops and therefore forage production must be adapted according to these new conditions. Resilient characteristics of forage, such as winter hardiness and ability to survive both flooding and droughts, have therefore become important attributes when choosing grass species for silage making.

In the Nordic countries, traditional grasses, such as meadow fescue (*Festuca pratensis* L.) and timothy (*Phleum pratense* L.), are well documented as grasses with high nutritive values [3,4], but they are not very drought tolerant [5]. As alternatives, there are some interesting species such as tall fescue (*Festuca arundinacea* Shreb) with high DM yield, drought tolerance, more tolerance to flooding and stronger winter hardiness the tall fescue hybrid festulolium (*Lolium multiforum* × *Festuca arundinacea*) [6], as well as reed canary grass (*Phalaris arundinacea* L) [7]. The herbage yields of these grasses are similar to or higher than yields of traditional grasses under normal conditions [8] and the yields can even be more superior under extreme weather conditions such as droughts and floods [6,7,9,10].

For alternative forage crops to be competitive in livestock systems, not only the herbage yields matter, but also their nutritive value and thereby possible influence on animal production levels needs to be considered. Tall fescue grass types have different cell wall structures than traditional grasses, such as timothy and meadow fescue, containing higher concentrations of hydroxycinnamic acids [11]. These acids prevent potentially digestible cell wall polysaccharides from being extensively digested in the rumen [12] and therefore limit ruminal fiber digestibility [13]. Feeding tall fescue grass types can therefore result in lower production levels than when using traditional grasses [14]. Feeding reed canary grass instead of traditional grasses, such as timothy and meadow fescue, can also result in lower feed intake due to its high fiber concentration [15] and content of the antinutritional alkaloids substances [7], giving the grass a low palatability resulting in low feed intake. Hence, when economic competitiveness of various forage crops is compared, the entire chain from cultivation inputs, through herbage yields and animals’ feed intake and production output needs to be taken into account, not the least in forage-based production systems. Such studies are rare, and to our knowledge, there is no such study comparing traditional and wet-and-drought-resistant grass species in northern Europe.

The aim of this study was to compare the profitability of forage-based cattle production of three intensity levels using diets composed of silages of traditional versus alternative wet-and-drought-resistant grass species in three regions of Sweden, each under three different weather conditions influencing harvest time and yield, based on recent research data.

The hypotheses were:

- Alternative grasses are relatively more competitive compared to traditional grasses under dry and wet weather conditions than under normal conditions due to relatively higher yield in the alternative grasses under extreme weather conditions.
- Alternative grasses are relatively more competitive compared to traditional grasses in extensive systems such as cow-calf production, than in intensive systems such as dairy production, due to the lower nutritive value in the alternative grasses.

## 2. Materials and Methods

### 2.1. Biological Data

Biological data used in this study were taken from three previous experiments conducted in southwestern Sweden comparing cattle production using silages made of traditional grasses (TR) and wet-and-drought-resistant grasses (WD). The studies included various animal categories, representing three levels of production intensity: dairy cows as high-intensive, beef breed bulls as semi-intensive, and beef suckler cows as low-intensive production systems. An overview of the studies is found below and in Table S1a–c, and details for dairy cows can be found in Sousa et al. [16], for beef bulls in Holmström et al. [17] and for beef cows in Jardstedt et al. [18].

Data on dairy cows were retrieved from a study conducted at Lantmännen Dairy Research Farm Nötcenter Viken, Falköping (58°17′ N, 13°55′ E) in 2016. Primiparous and multiparious dairy cows of Holstein and Swedish Red breeds were fed a total of mixed rations based on silage made of either timothy (TR) or tall fescue (WD) for two months in mid-lactation. Both grasses were harvested at a regular early date and a late date which occurred 11 and 17 days later than regular for tall fescue and timothy, respectively, altogether resulting in the basis of four experimental diets. All silages were complemented with the same pelleted cow compound feed with the same forage-to-concentrate ratio and all four diets were fed at ad libitum intake, therefore the consumption differed between diets but still, the ratio of forage to concentrate was the same. For the present study, experimental data were re-calculated to a 305-day lactation for a full year and all-year-calving, including grazing on temporary grasslands during summer whilst maintaining full indoor feed rations (Table S1a).

Data on slaughter, bulls of Continental beef breeds was compiled from a study conducted at Rådde Farm at the Rural Economy and Agricultural Society Sjuhärad, Länghem (57°61′ N, 13°26′ E) in 2021–2022. The bulls were monitored from weaning to slaughter at a target live weight of 680 kg, whilst kept indoors and fed silage of either meadow fescue (TR) or tall fescue (WD) at ad libitum intake, complemented with the same small amount of grain per head in the different groups. The experimental grasses were harvested at regular early dates. For an alternative with bulls fed late cut silages, simulated feed intake, live weight gain, and slaughter age (Table S1b) were estimated based on the chemical composition of grasses harvested 8 to 17 days after the regular early cut [19,20]. Carcass characteristics were assumed to be the same for bulls fed late cut as for bulls fed regular cut.

Data on beef cows were taken from a study conducted at Götala Beef and Lamb Research Centre, Swedish University of Agricultural Sciences, Skara (58◦42′ N, 13°21′ E) in 2013–2014. Multiparous, spring-calving Hereford cows were fed silages of meadow fescue—timothy (TR), festulolium (WD-f), or reed canary grass (WD-r) at ad libitum intake and as the only feed during pregnancy in winter. Due to the low nutritional requirement of dry beef cows, all forages were regularly harvested at late dates. Therefore, the chemical composition of silages cut at regular and late times was supposed to be the same. After calving, cows were grazed with their calves on semi-natural pastures during summer and the calves were weaned in autumn. The experimental data were somewhat modified in the present study to correspond to a full year for the beef cows including more days indoors where consumption before calving was estimated to be the same as they consumed daily during the experiment. Grazed grass was based on the estimation of the weaning weight of the calf and the weight of the cow before and after the grazing period (Table S1c).

Data on grass yields at regular harvest times were retrieved from Halling et al. [8] for all grasses fed to dairy cows and beef bulls, whereas yield data for late cut silage to these animals were taken from Nadeau and Hallin [21]. For regularly cut grasses fed to beef cows, yield data of meadow fescue—timothy and festulolium were retrieved from [8] and yield of reed canary grass from Palmborg [22]. Data on grass yield exposed to drought were retrieved from Joel et al. [23] for all grasses, except reed canary grass where yield data were taken from [7]. Except for the late cut grasses fed to beef bulls, where data on chemical composition were taken from Hallin et al. [19], all feed data were taken from the experimental study in question. The chemical composition was supposed to be the same during drought as under regular weather conditions.

### 2.2. Geographical Regions

**Figure 1:**
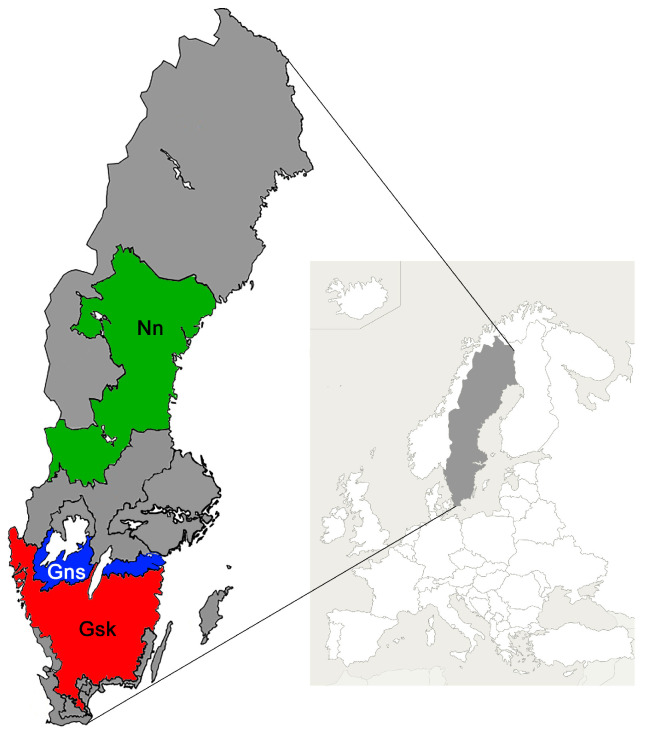
Localization of three geographical regions in Sweden; the forest districts in Götaland (Gsk), the plain districts in northern Götaland (Gns), and the lower parts of Norrland (Nn) [25]. Gsk and Gns are situated in the southern part with humid, warm temperate climate and Nn is situated in the north with cool summers.

Sweden is situated in the northern hemisphere, with a humid, snowy climate and cool summers in the northern part of the country and a humid, warm temperate climate with warm summers in the southern part [24]. The economic calculations of the three production systems with dairy cows, beef bulls, and beef cows described above were anticipated to occur in three alternative geographical regions in Sweden (Figure 1). These regions were chosen due to their varying natural and economic conditions and, for Sweden, large cattle populations [25,26]. The three regions were the forest districts in Götaland (Gsk), plain districts in northern Götaland (Gns), both in southern Sweden, and lower parts of Norrland (Nn) in northern Sweden, of which the first and last are located within less favored areas (LFA) with significantly less favorable field configurations and lower cost of arable land than Gns [25]. Annual precipitation and average temperature are in Gsk 730 mm and 6 °C, 600 mm and 7 °C in Gns, and 800 mm and 5 °C in Nn [2].

### 2.3. Weather Scenarios

Economic consequences of using TR or WD grasses in all three different cattle production systems in all three different geographic regions was calculated for three different weather scenarios, composing both of the historically normal climate and the coming climate with a forecasted increased frequency of extreme weather events [2]. Therefore, in addition to a reference scenario (Ref), based on present average conditions for grass yield, two other situations were calculated representing extremely wet weather with delayed harvest (Wet) and extremely dry conditions during cropping season (Dry). It was assumed that the Wet scenario resulted in a two-week delay of grass harvest (based on data 8–17 days delay) compared to the regular harvest time in the Ref scenario. This delay resulted in increased yield (Table S2) with higher fiber concentration, but lower organic matter digestibility and protein concentration (Table S1). The chemical composition of grasses fed to beef cows was assumed to be unchanged in the Wet scenario compared to the Ref scenario, due to that these grass cuts normally occur late and no further changes in grass yield or nutrition value were expected. The Dry scenario implied drought with 216 mm less precipitation resulting in 37% lower yield for TR and 26–35% lower yield for WD than in the Ref scenario [7,23]. When calculating the need for additional area of semi-natural pastures for beef cows in the Dry scenario, grazing low-opportunity cost land was assumed, meaning no extra cost or revenue was added for this land. No irrigation was assumed as this is not common in Swedish livestock farming. Except for the harvest yields and chemical composition of the grasses, as described above, all other conditions, volumes, and prices was constant in the basic calculation of all three weather scenarios.

### 2.4. Economical Calculations

The profitability of the described production combinations was defined as contribution margin (CM = contribution to common cost, risk, and profit), calculated as Σ revenues − Σ variable costs, which represents the money generated to cover common costs and profits using a modified model of Agriwise [27]. In the model, revenues include animal products and public payments and support [16,17,18,26,27,28,29,30]. In this case, revenues in animal production were milk, weaned calf, and part of the carcass for the dairy cow, full carcass for the beef bull, and weaned calf and part of the carcass for the beef cow. Farm support such as animal premium and support for milk production in Nn and also the fertilizer value of farm-yard manure were included (Table S3). Variable costs are costs associated with the production that will disappear if production ceases, e.g., animal purchase and feeds, whereas common costs include management that is common to all branches of production at the holding, such as planning, labor management, accounting and other administration (Table S3) [16,17,18,27,31,32,33,34,35]. Risk includes market risks, e.g., grossly lowered pay for animal products, biological risks, e.g., severely bad feed harvests, and political risks, e.g., lowered or abolished farm supports.

In this study, CM for all 54 combinations of grasses (TR, WD), animals (dairy cow, beef bull, beef cow), regions (Gsk, Gns, Nn), and scenarios (Ref, Wet, Dry) were computed. The calculations were made for herds with 100 dairy cows, 50 beef bulls slaughtered per year, and 50 beef cows, due to typical average Swedish herd sizes. Average prices for the years 2019–2023 were used in the calculations hence reflecting averages prices over a longer time [29,30].

Grass silage costs used in the contribution calculations were estimated for different field configurations in the different regions [36]. Gns was supposed to have large fields with a rectangular shape, while Gsk and Nn had small, scattered, and irregular fields (Table S2). The cost of grass silage and pastures was calculated as (Σ variable costs − Σ supports and payments)/net yield of silage/pasture. Variable costs of grass silage included machinery for a round bale system, labor costs [37], cropping costs [27,38], and opportunity cost of land, i.e., CM in autumn wheat and spring barley cropping in the different regions including support to single farm payment and LFA. Revenues from the silage and pasture included single farm payment, support to LFA, and for pasture revenues as well as agri-environmental payment to semi-natural pastures. All payments and supports were assumed to be constant across the weather scenarios (Table S4). Variable costs of pasture included topping, fencing, labor costs [27], and opportunity cost of grazed temporary grassland for the dairy cows in the same way as described above. The opportunity cost for permanent semi-natural pastures was calculated as rent cost (Table S4) [25]. Investment in a new building (Table S3) was supposed where the expense for the building (stanchion barns) was estimated from cost calculations, after investment support [31]. Labor demand per animal included all work associated with the animals during both indoor and grazing periods, but not work connected to feed cropping and maintenance of pasture (Table S3). Yearly labor demand in hours for dairy cows was taken from Agriwise [27]. Labor demand per reared beef bull was computed by using a model from Nelson [34] verified by [33]. Yearly labor demand for beef cows was estimated as 40 − 11.5 × log10(x) where x is number of beef cows, and subtracted by time for fencing, administration, and maintenance of buildings and machinery [32].

### 2.5. Sensitivity Analysis

In addition to the basic calculations, CM for one other situation was calculated for all combinations of production systems, geographical regions, and weather scenarios. In the basic calculation, the cost for arable land was set to the CM for the production of grain as the opportunity cost. In a sensitivity analysis, the cost for arable land was instead set to the average rental including free rent cost in the specific geographic region. The rental cost is lower than the opportunity cost, especially in Gns where CM in grain production is high. Rental cost is a relevant cost for land in parts of the region where the conditions are less suitable for grain production.

## 3. Results

### 3.1. Basic Calculation

The revenues in cattle enterprises are composed of two parts: payment for products and agri-environmental payments and supports. In this study, more than half of the revenues were derived from animal products in the high-intensive dairy and semi-intensive beef bull production, while for beef cows in low-intensive cow-calf production, a larger proportion of the revenues was derived from payments and supports (Tables S5–S7). For dairy cows in Nn a regional extra support for milk production increased the CM relative to other regions. The largest cost for dairy cows was winter feed (concentrate and silage), followed by costs for labor and replacement (Table S5). For beef bulls, the largest costs were the purchase of calf and costs for silage and building (Table S6) and for beef cows, the largest cost was silage, followed by labor and building costs (Table S7).

#### 3.1.1. Normal Weather Conditions

**Figure 2:**
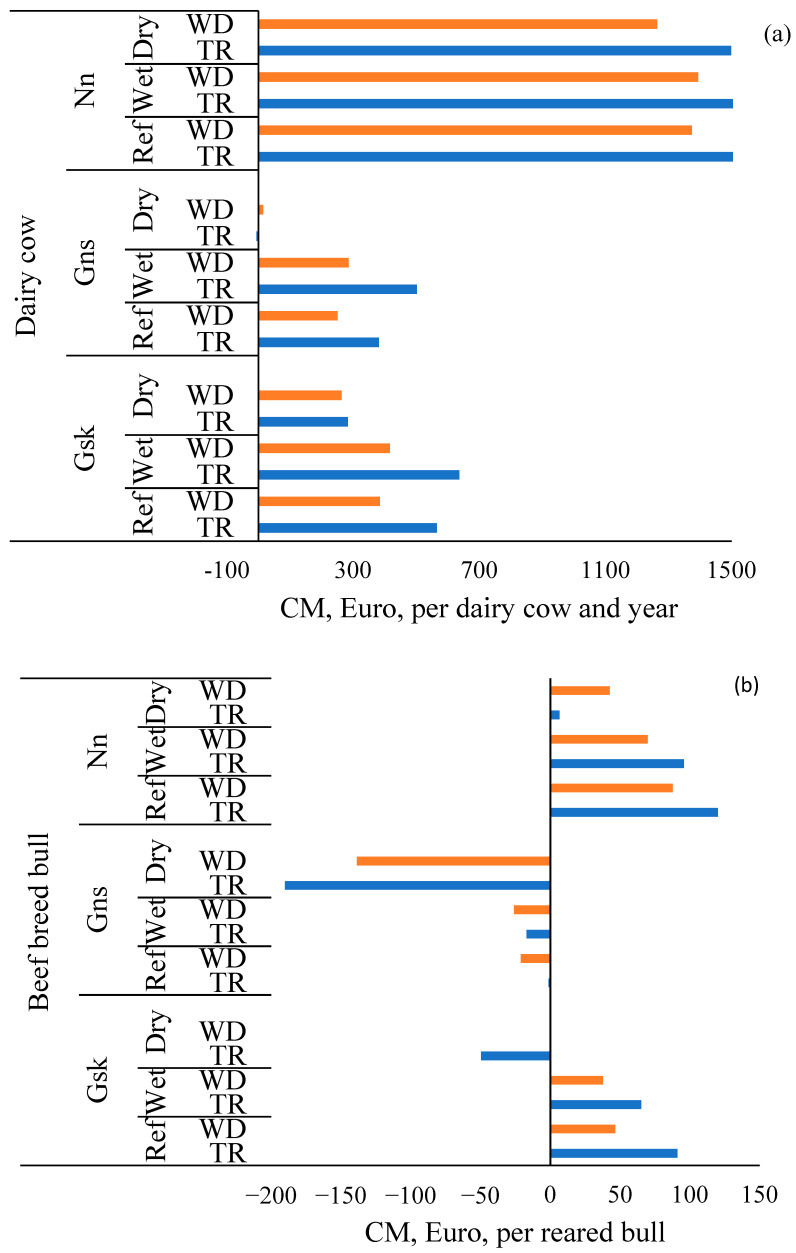

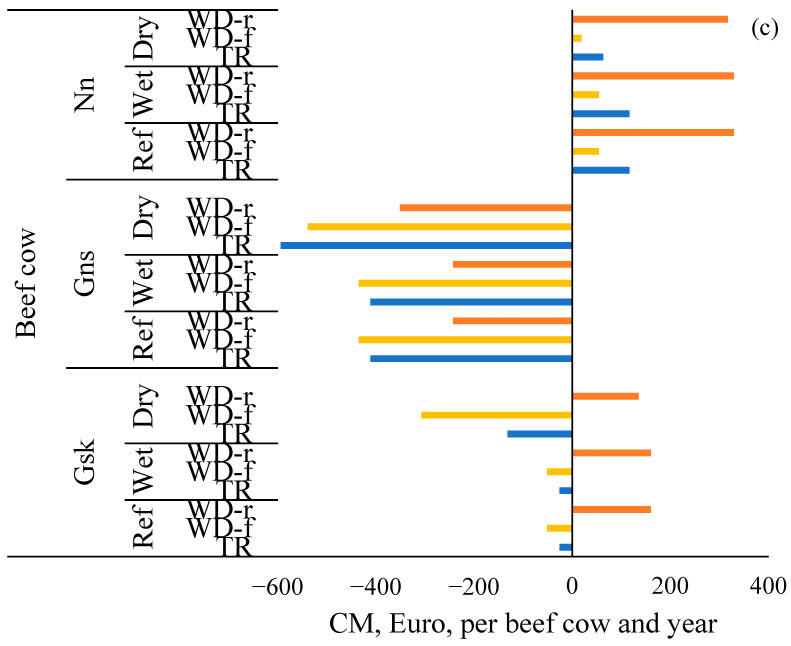
Basic calculation of contribution margin (CM = contribution to common cost, risk, and profit) in three different cattle production systems; (**a**) dairy cow, (**b**) beef breed bulls, and (**c**) beef cow, fed silage of traditional (TR) or wet-and-drought-resistant (WD) grasses in forest districts Gsk, plain districts Gns, and northern districts Nn in Sweden, in three scenarios: reference (Ref), wet (Wet), and dry (Dry). TR was timothy (dairy cow), meadow fescue (bull), and meadow fescue-timothy (beef cow), WD was tall fescue (dairy cow and bull), WD-f festulolium (beef cow), and WD-r reed canary grass (beef cow). Expressed as Euro per cow and year or per reared bull.

For dairy cows and beef bulls, TR was more competitive than WD under normal weather conditions in all regions (Figure 2a,b, Tables S5 and S6). For beef cows, WD-r was more competitive than TR and WD-f in all regions, where TR had somewhat higher CM than WD-f (Figure 2c, Table S7).

#### 3.1.2. Dry Weather Conditions

Profitability for all cattle systems was impaired in the Dry scenario compared to the Ref scenario (Figure 2). The decrease in CM was due to a higher cost for winter feed, which affected alternatives with TR more than alternatives with WD.

For dairy cows in the Dry scenario, TR still was more competitive than WD in Nn, whereas CM were similar for the two grasses in Gsk and Gns (Figure 2a, Table S5). Contrary, for beef bulls, WD gave higher CM than TR in all regions in this scenario (Figure 2b, Table S6). For beef cows in the Dry scenario, WD-r was more competitive than TR and WD-f in all regions (Figure 2c, Table S7). The TR had higher CM than WD-f in Gsk and Nn (Figure 2c, Table S7).

#### 3.1.3. Wet Weather Conditions

In the scenario with delayed cutting time due to wet weather conditions, CM across production systems and regions was rather unchanged compared to CM in the Ref scenario (Figure 2).

The TR still was more competitive than WD for dairy cows and beef bulls in all regions (Figure 2a,b, Tables S5 and S6). However, the difference in CM for the two grasses was minor for beef bulls in Gns. For beef cows, the regular and late harvest times were the same and hence the CM in the Wet scenario was the same as in the Ref scenario with WD-r being more competitive than TR and WD-f in all regions (Figure 2c, Table S7).

#### 3.1.4. Fluctuating Weather Conditions

For dairy cows in Gns, WD resulted in higher CM than TR only when every year was like the Dry scenario (Table S8a). Not even with yearly droughts, WD was more profitable than TR for dairy cows in Gsk and Nn. Which grass species resulted in the highest CM for beef bulls differed between the weather scenarios (Table S8b). With mainly normal weather conditions, TR resulted in higher CM for beef bulls than WD did. However, when Dry scenarios occurred every second year or more, WD resulted in a higher CM than TR (Table S8b). For beef cows, WD-r had higher CM than TR had, independent of proportion of years with normal, wet, and dry weather conditions or region (Table S8c).

### 3.2. Sensitivity Analysis with Rent Cost for Land

Compared to the basic calculation in the Ref scenario, the sensitivity analysis using the lower rent cost including free rent cost, instead of opportunity cost for land, drastically decreased the production cost of the silages (Table S9). Across cattle system, region, and scenario, the silage cost decreased between 0.021 and 0.102 EUR/kg DM for TR and 0.019 and 0.074 EUR/kg DM for WD (Table S9). The decreased silage cost and grass grazed on temporary grasslands (Table S10) positively affected CM of the animal production, in general more for TR than WD (Table S11). The cost of the silages differed more between the basic calculation and the sensitivity analysis in Gns than in Gsk and Nn, resulting in a larger effect on the CM in the former region. Taken together, the lower cost of the silages had the most positive effect on CM for dairy cows in Gns, especially for TR (Table S11a). Changes in silage cost in the sensitivity analysis were rather similar between TR and WD and did therefore generally not affect the ranking in CM of the two grasses. The only exception was for dairy cows in Gns in the Dry scenario, where CM for WD was slightly higher than CM for TR in the basic calculation, whilst TR was superior in the sensitivity analysis (Table S11a).

## 4. Discussion

The calculations for the different scenarios Ref, Wet, and Dry were based on studies of normal, wet, and dry weather conditions in the Swedish climate of today. Therefore, the results are mainly relevant in the short term before the ongoing climate changes make the variations in weather more extreme. Nevertheless, results from this study can indicate the economic competitiveness of different grasses also under continued climate change.

According to the economic calculations, the relative competitiveness of the grasses varied along with the intensity level of the cattle production systems. In high-intensive dairy production, TR mainly shows a higher profitability than WD, except for the Dry scenario in Gns where WD was slightly more profitable (Table S5). The superiority of the TR was due to a higher milk yield for cows fed TR than for cows fed WD, but drought in Gns, with lower grass yields and higher opportunity cost of land, affected TR more negatively than WD. On the other hand, low-intensively reared beef cows had higher profitability when offered WD-r than WD-f or TR. Because of the low nutrient requirement of beef cows during early gestation, WD-r is well suited to this animal category [18]. In the study by Jardstedt et al. [18] all beef cows became pregnant the forthcoming summer except two fed WD-f. The number of beef cows in that study was small and the study was just one year, hence it was difficult to draw conclusions about the effect of diet on cow pregnancy rate. However, lower reproductive performance is something that can have a substantial impact on the beef cow system and profitability. Therefore, one should be observant of possible effects on fertility when considering new diets. The cost of producing WD-r was in this study the lowest of all grasses produced in Gsk and Nn. When using rent cost instead of opportunity cost for arable land, WD-r was the cheapest grass also in Gns. Consequently, WD-r was the most profitable silage for feeding beef cows in all weather scenarios. For semi-intensive beef breed bulls, TR was the most profitable grass in the Ref and Wet scenarios, whereas WD was more competitive in the Dry scenario. The reason for the shift in most profitable grass between scenarios for the bulls, is the magnitude of the silage cost in this production, being the second largest cost after the purchase of beef calf. Hence, the silage cost has a large impact on the economic result in beef bull production, which was also shown by Holmström et al. [39] comparing different cattle systems across Sweden.

The profitability of all cattle systems was impaired in the Dry scenario compared to the Ref scenario. This was due to a lower yield and thereby higher cost per kg DM silage, thus higher feed costs. Drought affects the yield of TR more than the yield of WD [7,8,22,23] and the price is thereby also more affected for TR than for WD. Furthermore, drought will have an even larger impact on CM if the silage production does not cover the needs of the farm. To compensate for the lack of silage, animals must be fed more concentrate, probably at a higher expense than supposed in this study. Comparing changes in CM for the cattle systems examined shows that CM for beef bulls decreased more than CM for dairy cows when changing from the Ref to the Wet scenario. Hence, bulls were most sensitive to a delayed grass harvest in the Wet scenario, while dairy cows had a higher CM in this scenario. The difference between the cattle systems is due to the fact that the lower cost of silage in Wet, compared to Ref, did not compensate for the longer rearing time for bulls, whereas for dairy cows, the changes in revenues for milk and cost of concentrate was compensated by lower cost of silage.

It is impossible to know the details of future weather. Nevertheless, researchers have stated a direction for the forecasted climate changes, with higher temperatures and more frequent extreme weather events to be expected [1]. We can expect shorter winters and longer vegetation periods, combined with more extreme weather such as flooding, drought, and heavy rainfalls in Sweden [2], probably with even higher amplitudes than calculated for Wet and Dry scenarios in this study. Therefore, we have calculated with rain and drought for more years than one to see if there was a break-even. The changing weather will affect cultivated crops in different ways. For instance, TR timothy will be stressed during waterlogging and higher autumn temperatures will affect the winter hardiness negatively, which in turn can reduce winter survival [40]. Higher temperatures during the growing season will stimulate the grasses to mature faster, accompanied by a rapid decline in forage digestibility [3,41]. The adaptive capacities vary among grass species, where, e.g., WD-f has a highly diversified germplasm base and thereby an enhanced capacity to adapt to climate change [5].

The relative competitiveness between the studied grasses will change if the weather results in even more severe flooding or drought. Therefore, it could most likely be expected that WD’s ability to both survive in waterlogged land and to grow with less water will, in the future, be even more important for cattle production. The establishment of new leys for grass production in our scenarios was not affected by weather situations but can be of great importance with a changed climate. Thereby changes in cultivation approaches may be made. This study shows that animal production CM is not much impaired by a delayed cut of grass, but if the rainfall affects the number of days grazing, or results in even lower nutritional value, CM might be affected. As stated by Shalloo et al. [42], more rain would have a very negative effect on profitability in pasture-based dairy production, which might not be economically sustainable at all due to less availability of low-cost grazing. Although the CM of the cattle systems was not very much affected by the late cut of grass in the Wet scenario of this study, the CM was very impaired by drought in the Dry scenario. In a study in the Spanish Pyrenees, the economic margin of beef cow enterprises decreased drastically because of drought. This was due to less grazing, which affected the forage cost, and the drought might also have affected calf weaning weight negatively [43]. In the present study, neither lower yield on pasture nor lower weaning weight was supposed in the Dry scenario, but still, there was a dramatic reduction of CM in the Dry scenario compared to Ref. Therefore, finding grasses that can withstand drought and still be suitable for a high animal production level is important. WD in this study shows a higher yield than TR in the Dry scenario. Thereby WD was more competitive than TR during drought. If the drought had been even more severe than in the Dry scenario, the decrease in TR yield would only have had to be 0.2 tons/ha before WD would be on the same CM level as TR for dairy cows in Gsk. On the other hand, in Nn, the yield of TR would have had to decrease by 1.4 tons/ha before WD could compete with TR in dairy production. Furthermore, for beef bulls, WD gave a higher CM than TR when drought occurred every second year or more frequently.

For semi-intensively fed beef bulls, WD was more competitive than TR in the Dry scenario. WD was more competitive than TR only in one case for high-yielding dairy cows. Due to higher yield, WD had a lower cost for silage production resulting in a higher CM although milk yield and carcass weight gain were higher when feeding TR. Hence, higher CM for cattle fed WD compared to TR was even more pronounced with a higher frequency of dry years.

This study showed WD-r gave the highest CM for low-intensive beef cows due to the high fiber content of the silage and hence a lower intake. However, WD gave lower CM than TR for high-intensive dairy cows due to lower revenue from milk, except in the Dry scenario in Gns when CM for WD was slightly higher than CM for TR. For beef bulls, CM was higher for WD than for TR in the Dry scenario. Consequently, it seems like high-intensive dairy cows are more sensitive to the lower digestibility of WD than semi-intensive beef bulls and low-intensive beef cows.

Forage quality within grass species can be affected by different factors, such as location, weather, and maturity at harvest and it is well reported in the literature [44]. However, the effect of forage quality between grass species on animal performance is not completely understood. To our knowledge, there is only one group that evaluated the effects of grass species and harvest date on the performance of dairy cows in Nordic conditions. Sousa et al. [18] compared timothy and tall fescue (in the present study defined as TR and WD) harvested at regular and late maturity; Sousa et al. [45] compared TR and WD harvested at regular maturity from the first and second cut, and Sousa et al. [46] compared TR and WD harvested at very early and early dates. Results were consistent across all studies, where even with fiber components increasing with maturity stage, the performance of dairy cows was only affected by grass species. In conclusion, milk yield or feed efficiency was greater in cows fed TR-based diets compared to cows fed WD-based diets, regardless of harvest date.

Although TR, with its lower grass yield than WD, had a higher cost per kg of DM in the Ref scenario, milk yield and carcass weight gain in animal production had a greater effect on the CM, especially in dairy production. For dairy cows, TR gave CM that were 130 to 321 EUR/cow and a year higher than WD, depending on region. Smaller differences in CM were found for beef bulls fed TR and WD, varying between 20 to 44 EUR/reared bull in favor of TR. The cost of silage was higher for TR than for WD in Gns and Nn, whilst the cost was more equivalent for silages in Gsk. Beef cow operations cut the grass late with accompanying high grass yields and low nutritional values, resulting in only small differences between the cost for TR and WD-f in all regions. Due to a very long crop rotation time span and a high yield, WD-r had a lower, or almost lower, silage production cost than all other grasses. The largest differences in silage cost were found in the Dry scenario, where WD fed to dairy cows and beef bulls had the lowest cost due to higher yield than TR. For beef cows, WD-r had the lowest cost except in Gns, calculated with the opportunity cost for arable land, and, interestingly, WD-f had a higher cost than TR in Gsk.

Integrating the cost of fodder production and animal production into economic calculations may not result in the production models with the highest animal production levels also having the highest CM [39,47]. The digestibility of the crop is not the only important aspect, also the cost of production of the silage is crucial. The yield is the most defining factor for the cost, with a higher yield decreasing the production cost of the crop [27,48], as is also seen in this study. Even if a grass species gives the highest animal production level, it can be too expensive to produce, and the profitability of the animal production might thereby be lower than if fed another more production-limiting silage. In the present study, it was shown that under the predicted weather conditions in the Ref and Wet scenarios, the expected animal production results also resulted in the highest CM for both dairy cows, beef bulls, and beef cows, but there were differences depending on region and weather scenarios. This variation in CM was mainly due to large differences among the studied regions, with high grass yields but also high cost for arable land and a lack of LFA compensation in the southern plains Gns, compared to the forest and northern districts (Gsk and Nn) [26]. Agri-environmental payments and supports affected the results creating higher CM for all calculated animal production systems in Nn. In Nn, longer transports and smaller fields than in Gsk and Gns were assumed. The assumed differences might of course have been too small, and distances and/or scattered field shapes might have had larger effects and affect the costs of silage even more. Also, the opportunity cost of land heavily affects the cost of silage. This is shown when comparing the costs of silages between different regions, especially Gns compared to Gsk and Nn. The effect of the opportunity cost becomes very clear in the sensitivity analysis when the opportunity cost is changed to the rent cost of land. An alternative, and sometimes more profitable, use of semi-natural pastures in southern Sweden (Gsk and Gns) could be to plant spruce on the most fertile areas instead of maintaining grazing [49] which, if there was a lack of semi-natural pastures, would affect the profitability of beef cow enterprises negatively in those regions. To keep cattle in areas with an excellent grain production capacity could result in losses of profitability, compared to cash-crops, especially for beef cows. Nevertheless, also in regions with mainly high-productive arable land, there are less-yielding areas suitable for different cattle production systems.

In Gns and Nn, the cost of silage differed when using opportunity cost or rent cost for arable land in which silages for beef cows had the lowest cost. In the plains of Gns, WD-f was as expensive as TR in the Ref and Wet scenarios, when cost calculation was based on rent cost, whereas TR had a higher cost than WD-f when using opportunity cost in calculations. This is a mixed effect of yield and cost for land, where a lower yield and high opportunity cost made up a higher price. In Nn in the Dry scenario, WD-f became more expensive than TR when using rent cost for arable land. However, opportunity cost made WD-f cheaper than TR, but still, WD-r was the most affordable grass for beef cows. The low yield in the Dry scenario and the cost of growing WD-r in Nn being just a bit over the agri-environmental payments and supports, resulted in only a minor decrease in silage cost when changing from opportunity cost to rent cost for beef cows in Nn. In Swedish beef cow herds, WD-r is not a common fodder, but the result from this study shows there are possibilities to increase the low profitability in beef cow enterprises by feeding WD-r.

Choosing the best species and mixtures for temporary grasslands is a way to utilize opportunities and meet challenges in the ongoing climate changes. Grasslands that can combine good yield, forage quality, yield stability, and survival of plants also under new weather conditions are essential for a sustainable future forage production. There is a need for more research on using grass–clover mixtures in different weather scenarios as this is the most common forage cropping strategy on Swedish farms.

## 5. Conclusions

The calculation shows that under normal and wet weather conditions, TR gave higher CM than WD when fed to dairy cows and beef bulls, regardless of geographical region. Under dry weather conditions, WD was more competitive than TR in beef bull production, but in dairy production, WD only gave a slightly higher CM in one out of the three studied regions. For beef cows, WD-r always resulted in the highest CM, regardless of weather conditions and region. Changing from normal to wet weather conditions impaired CM of the beef bulls more than CM of the dairy and beef cows. Silage production cost was generally lower for WD than for TR, where the largest difference in cost was under dry weather conditions and when using rent cost instead of opportunity cost for the land. The silage cost was also affected by region. It was concluded that WD can be an alternative to TR in low-intensive cattle systems, for example, being fed to beef cows. However, even with a high frequency of dry weather conditions, WD is rarely competitive over TR in high-intensive cattle systems, where its lower production cost is outweighed by its negative effect on the yield of animal products.

## Data Availability

The original contributions presented in the study are included in the article/Supplementary Materials; further inquiries can be directed to the corresponding author.

## Supplementary Materials

The following supporting information can be downloaded at: https://www.mdpi.com/article/10.3390/ani15030295/s1. Table S1: **a**: Grass silage characteristics, yearly consumption of grass silage, concentrates and grazed grass, and milk yield per dairy cow fed a diet based on silage of traditional (TR) timothy or wet-and-drought-resistant (WD) tall fescue from regular or late cut harvests; **b**: Grass silage characteristics, consumption of grass silage and concentrates, carcass weight and slaughter age per reared slaughter beef bull fed a diet based on silage of traditional meadow fescue (TR) or wet-and-drought-resistant tall fescue (WD) from regular or late cut harvests; **c**: Grass silage characteristics, yearly consumption of grass silage and grazed grass, and calf weaning weight per beef cow winter-fed with silage of traditional meadow fescue-timothy (TR), wet-and-drought-resistant festulolium (WD-f), and wet-and-drought-resistant reed canary grass (WD-r) from regular or late cut harvests; Table S2: Description of three geographical regions, showing their type, field configuration, farm size for calculation of opportunity cost, and yields of harvested silage and grazed temporary grasslands, expressed in ton dry matter (DM) per hectare (ha) used when comparing the profitability of various combinations of cattle production systems, grass silages (timothy, meadow fescue, and timothy-meadow fescue are traditional grasses (TR) and tall fescue, festulolium, and reed canary grass are alternative wet-and-drought-resistant grasses (WD)), and weather scenarios (historically normal weather, Reference (Ref), wet weather with a two-week delay of harvest (Wet), and dry weather with 216 mm lower precipitation than normal (Dry)); Table S3: Method and data used for calculating revenues when comparing the profitability of various combinations of cattle production systems, grass silages, weather scenarios, and geographical regions in Sweden; calculated as average price 2019–2023 for milk, calf, carcass, calf purchase, concentrate, and labor. Other prices are collected from sources with prices originating from different years. Those sums are index calculated to the same average price level as above by using series of indexes for different means of production; 11.53 SEK = 1 Euro; ha = hectare; Table S4: Method and data used for calculating revenues and costs in silage and pasture production when comparing the profitability of various combinations of cattle production systems, grass silages, weather scenarios, and geographical regions in Sweden; calculated as average price 2019–2023 for seed, fertilizer, machinery pasture, fence, labor, and opportunity cost. Other costs are collected from sources with prices originating from different years. Those sums are index calculated to the same average price level as above by using series of indexes for different means of productiona (Swedish Board of Agriculture, 2023a); 11.53 SEK = 1 Euro; ha = hectare; Table S5: **a**: Contribution margin (= contribution to common cost, risk, and profit) in a herd of 100 dairy cows located in forest districts of Götaland (Gsk), where cows are fed grass silages of either traditional (TR) timothy or wet-and-drought-resistant (WD) tall fescue under three different weather scenarios; historically normal weather, Reference (Ref), wet weather with a two-week delayed grass cut compared to Reference (Wet); and dry weather with 216 mm lower precipitation and hence 26–37% lower herbage yield than in Ref (Dry), Euro per cow and year; **b**: Contribution margin (= contribution to common cost, risk, and profit) in a herd of 100 dairy cows located in plain districts in northern Götaland (Gns), where cows are fed grass silages of either traditional (TR) timothy or wet-and-drought-resistant (WD) tall fescue under three different weather scenarios; historically normal weather, Reference (Ref), wet weather with a two-week delayed grass cut compared to Reference (Wet); and dry weather with 216 mm lower precipitation and hence 26–37% lower herbage yield than in Ref (Dry), Euro per cow and year; **c**: Contribution margin (= contribution to common cost, risk, and profit) in a herd of 100 dairy cows located in lower parts of Norrland (Nn), where cows are fed grass silages of either traditional (TR) timothy or wet-and-drought-resistant (WD) tall fescue under three different weather scenarios; historically normal weather, Reference (Ref), wet weather with a two-week delayed grass cut compared to Reference (Wet); and dry weather with 216 mm lower precipitation and hence 26–37% lower herbage yield than in Ref (Dry), Euro per cow and year; Table S6: **a**: Contribution margin (= contribution to common cost, risk, and profit) in a herd of 50 reared beef breed bulls per year located in forest districts of Götaland (Gsk), where bulls are fed grass silages of either traditional (TR) meadow fescue or wet-and-drought-resistant (WD) tall fescue under three different weather scenarios; historically normal weather, Reference (Ref), wet weather with a two-week delayed grass cut compared to Reference (Wet); and dry weather with 216 mm lower precipitation and hence 26–37% lower herbage yield than in Ref (Dry), Euro per reared bull; **b**: Contribution margin (= contribution to common cost, risk, and profit) in a herd of 50 reared beef breed bulls per year located in plain districts in northern Götaland (Gns), where bulls are fed grass silages of either traditional (TR) meadow fescue or wet-and-drought-resistant (WD) tall fescue under three different weather scenarios; historically normal weather, Reference (Ref), wet weather with a two-week delayed grass cut compared to Reference (Wet); and dry weather with 216 mm lower precipitation and hence 26–37% lower herbage yield than in Ref (Dry), Euro per reared bull; **c**: Contribution margin (= contribution to common cost, risk, and profit) in a herd of 50 reared beef breed bulls per year located in lower parts of Norrland (Nn), where bulls are fed grass silages of either traditional (TR) meadow fescue or wet-and-drought-resistant (WD) tall fescue under three different weather scenarios; historically normal weather, Reference (Ref), wet weather with a two-week delayed grass cut compared to Reference (Wet); and dry weather with 216 mm lower precipitation and hence 26–37% lower herbage yield than in Ref (Dry), Euro per reared bull; Table S7: **a**: Contribution margin (= contribution to common cost, risk, and profit) in a herd of 50 beef cows located in forest districts of Götaland’s (Gsk) lower parts, where cows are fed grass silages of either traditional (TR) meadow fescue-timothy, wet-and-drought-resistant (WD-f) festulolium, or wet-and-drought-resistant (WD-r) reed canary grass under three different weather scenarios; historically normal weather, Reference (Ref), wet weather with a two-week delayed grass cut compared to Reference (Wet); and dry weather with 216 mm lower precipitation and hence 26–37% lower herbage yield than in Ref (Dry), Euro per cow and year; **b**: Contribution margin (= contribution to common cost, risk, and profit) in a herd of 50 beef cows located in plain districts in northern Götaland’s (Gns) lower parts, where cows are fed grass silages of either traditional (TR) meadow fescue-timothy, wet-and-drought-resistant (WD-f) festulolium, or wet-and-drought-resistant (WD-r) reed canary grass under three different weather scenarios; historically normal weather, Reference (Ref), wet weather with a two-week delayed grass cut compared to Reference (Wet); and dry weather with 216 mm lower precipitation and hence 26–37% lower herbage yield than in Ref (Dry), Euro per cow and year; **c**: Contribution margin (= contribution to common cost, risk, and profit) in a herd of 50 beef cows located in lower parts of Norrland (Nn), where cows are fed grass silages of either traditional (TR) meadow fescue-timothy, wet-and-drought-resistant (WD-f) festulolium, or wet-and-drought-resistant (WD-r) reed canary grass under three different weather scenarios; historically normal weather, Reference (Ref), wet weather with a two-week delayed grass cut compared to Reference (Wet); and dry weather with 216 mm lower precipitation and hence 26–37% lower herbage yield than in Ref (Dry), Euro per cow and year; Table S8: **a**: Contribution margin (= contribution to common cost, risk, and profit) in calculation with varying proportions of years with different weathers; historically normal weather, Reference (Ref), wet weather with delayed harvest (Wet), and dry weather with lower grass yield (Dry), when feeding grass silages of either traditional (TR) timothy or wet-and-drought-resistant (WD) tall fescue in a herd with 100 dairy cows, located in forest districts (Gsk), plain districts (Gns), or northern districts (Nn) of Sweden, Euro per cow and year; **b**: Contribution margin (= contribution to common cost, risk, and profit) in calculation with varying proportions of years with different weathers; historically normal weather, Reference (Ref), wet weather with delayed harvest (Wet), and dry weather with lower grass yield (Dry), when feeding grass silages of either traditional (TR) meadow fescue or wet-and-drought-resistant (WD) tall fescue, in a herd with 50 slaughtered beef breed bulls per year located in forest districts (Gsk), plain districts (Gns), or northern districts (Nn) of Sweden, Euro per reared bull; **c**: Contribution margin (= contribution to common cost, risk, and profit) in calculation with varying proportions of years with different weathers; historically normal weather, Reference (Ref), wet weather with delayed harvest (Wet), and dry weather with lower grass yield (Dry), when feeding grass silages of either traditional (TR) meadow fescue-timothy, wet-and-drought-resistant festulolium (WD-f), or reed canary grass (WD-r) in a herd with 50 beef suckler cows located in forest districts (Gsk), plain districts (Gns), or northern districts (Nn) of Sweden, Euro per cow and year; Table S9: Cost for silage production in a basic calculation and a sensitivity analysis with rent cost instead of opportunity cost for arable land in herds with 100 dairy cows, 50 beef breed bulls, or 50 beef suckler cows fed grass silages of either traditional (TR) grasses (timothy for dairy cow, meadow fescue for bull, meadow fescue-timothy for beef cow) or wet-and-drought-resistant (WD) grasses (tall fescue for dairy cow and bull, festulolium (f) or reed canary grass (r) for beef cow) in forest districts (Gsk), plain districts (Gns), and northern districts (Nn) of Sweden in three different weather scenarios; historically normal weather, Reference (Ref), wet weather with delayed harvest (Wet), and dry weather with lower grass yield (Dry). Euro per kg dry matter; Table S10: Cost for grazed herbage on temporary grasslands in a basic calculation and a sensitivity analysis with rent cost instead of opportunity cost for land herds with 100 dairy cows in forest districts (Gsk), plain districts (Gns), and northern districts (Nn) of Sweden in three different weather scenarios; historically normal weather, Reference (Ref), wet weather with delayed harvest (Wet), and dry weather with lower grass yield (Dry); Table S11: **a**: Contribution margin (= contribution to common cost, risk, and profit) in a basic calculation and a sensitivity analysis with rent cost instead of opportunity cost for arable land, in a herd with 100 dairy cows fed grass silages of either traditional (TR) timothy or wet-and-drought-resistant (WD) tall fescue located in forest districts (Gsk), plain districts (Gns), or northern districts (Nn) of Sweden in three different weather scenarios; historically normal weather, Reference (Ref), wet weather with delayed harvest (Wet), and dry weather with lower grass yield (Dry), Euro per cow and year; **b**: Contribution margin (= contribution to common cost, risk, and profit) in a basic calculation and a sensitivity analysis with rent cost instead of opportunity cost for arable land in a herd with 50 slaughtered beef breed bulls per year fed grass silages of either traditional (TR) meadow fescue or wet-and-drought-resistant (WD) tall fescue located in forest districts (Gsk), plain districts (Gns), and northern districts (Nn) of Sweden in three different weather scenarios; historically normal weather, Reference (Ref), wet weather with delayed harvest (Wet), and dry weather with lower grass yield (Dry), Euro per reared bull; **c**: Contribution margin (= contribution to common cost, risk, and profit) in a basic calculation and a sensitivity analysis with rent cost instead of opportunity cost for arable land in a herd with 50 beef suckler cows fed grass silages of either traditional (TR) meadow fescue-timothy, wet-and-drought-resistant festulolium (WD-f), or reed canary grass (WD-r) in forest districts (Gsk), plain districts (Gns), and northern districts (Nn) of Sweden in three different weather scenarios; historically normal weather, Reference (Ref), wet weather with delayed harvest (Wet), and dry weather with lower grass yield (Dry), Euro per cow and year.
