# Supplementary material for: In a Changing World—An Economical Comparison Between Traditional and Wet-And-Drought-Resistant Grasses in Swedish Cattle Production Under Different Weather Scenarios
Source: Animals (Basel). 2025 Jan 21;15(3):295. doi: 10.3390/ani15030295 (PMC11816082; doi:10.3390/ani15030295)
Supplement: Supplementary file 1 [file animals-15-00295-s001.zip › animals-3414972-supplementary.pdf]

Supplementary materials

Table S1a. Grass silage characteristics, yearly consumption of grass silage, concentrates and grazed grass, and milk yield per dairy cow fed a diet based on silage of traditional (TR) timothy or wet-and-drought-resistant (WD) tall fescue from regular or late cut harvests. Data of regular cut is used in Reference and Dry scenarios and data from late cut in Wet scenario, NE = net energy, ECM = energy corrected milk

	Dairy cows[a]			
	Regular cut		Late cut	
	TR	WD	TR	WD
Grass silage				
NDF, g/kg DM	448	484	518	478
NE, MJ/kg DM	7.64	7.43	6.96	7.29
CP[a], g/kg DM	204	206	157	193
Animal				
Grass silage, ton DM	3.38	2.78	2.78	3.02
Concentrate, ton	4.51	3.93	3.71	4.02
Grazed herbage[b], ton DM	0.12	0.12	0.12	0.12
Milk yield, kg	11 377	9 852	10 400	9 882
Milk yield, kg ECM	11 468	10 218	10 614	10 431

Reference

[a] Sousa, D. O., Murphy, M., Hatfield, R., & Nadeau, E., 2021. Effects of harvest date and grass species on silage cell wall components and lactation performance of dairy cows. *Journal of Dairy Science*, *104*(5), 5391–5404. https://doi.org/10.3168/jds.2020-19362

[b]Agriwise, 2023. Agriwise - Smart kalkylering. https://www.agriwise.se/web (accessed 18 January 2024).

Table S1b. Grass silage characteristics, consumption of grass silage and concentrates, carcass weight and slaughter age per reared slaughter beef bull fed a diet based on silage of traditional meadow fescue (TR) or wet-and-drought-resistant tall fescue (WD) from regular or late cut harvests. Data of regular cut is used in Reference and Dry scenarios and data from late cut in Wet scenario, NE = net energy.

	Beef bulls			
	Regular cut[a]		Late cut[b,c]	
	TR	WD	TR	WD
Grass silage				
NDF, g/kg DM	500	488	509	493
NE, MJ/kg DM	6.04	6.09	5.78	5.78
CP, g/kg DM	166	173	148	151
Animal				
Grass silage, ton DM	1.83	1.90	1.94	1.97
Concentrate, ton	0.74	0.69	0.80	0.74
Carcass weight[a], kg	380	370	380	370
Age at slaughter, month	16.4	15.9	17.2	16.5

Reference

[a]Holmström K., Sousa D. O., and Hessle A., 2024. Productive Performance of beef bulls fed tall fescue silage or meadow fescue silage and complemented with cereal grains. Acta Agriculturae Scandinavica, Section A — Animal Science 2024, 1–5, doi:10.1080/09064702.2024.2387581.

[b]Hallin O., Holmström K., and Nadeau E., 2023. Vallfröblandningar anpassade till antal skördar – Forage seed mixtures adopted to number of harvests. Department of Animal and Health, Swedish University of Agriculture Sciences, Skara. (62).

[c]Volden H., 2011. NorFor-The Nordic feed evaluation system. EAAP publications. Wageningen Academic Publishers, Wageningen. (130).

Table S1c. Grass silage characteristics, yearly consumption of grass silage and grazed grass, and calf weaning weight per beef cow winter-fed with silage of traditional meadow fescue-timothy (TR), wet-and-drought-resistant festulolium (WD-f), and wet-and-drought-resistant reed canarygrass (WD-r) from regular or late cut harvests. Data of regular cut is used in Reference and Dry scenarios and data from late cut in Wet scenario, although no diffferences in silage chemical composition was assumed for beef cows, NDF = neutral detergent fibre, NE = net energy. Amount of feeds vary among regions (Gsk = forest district of Götaland, Gns = plain districts of Götaland, Nn = lower part of Norrland) due to varying length of grazing season

	Beef cow[a]					
	Regular cut			Late cut		
	TR	WD-f	WD-r	TR	WD-f	WD-r
Grass silage						
NDF, g/kg DM	576	543	648	576	543	648
NE, MJ/kg DM	5.8	6.4	5.2	5.8	6.4	5.2
CP, g/kg DM	111	97	130	111	97	130
Animal						
Grass silage, ton DM						
Gsk	2.32	2.50	1.65	2.32	2.50	1.65
Gns	2.32	2.50	1.65	2.32	2.50	1.65
Nn	2.77	2.99	1.97	2.77	2.99	1.97
Grazed herbage, ton DM						
Gsk	2.22	2.13	2.43	2.22	2.13	2.43
Gsn	2.22	2.13	2.43	2.22	2.13	2.43
Nn	1.81	1.72	2.01	1.81	1.72	2.01
Calf weaning weight, kg	317	316	312	317	316	312

Reference

[a]Jardstedt M., Nadeau E., Olaf Nielsen M., Nørgaard P., and Hessle A., 2019. The effect of feeding roughages of varying digestibility prepartum on energy status and metabolic profiles in beef cows around parturition. Animals (3)

Table S2. Description of three geographical regions, showing their type, field configuration, farm size for calculation of opportunity cost, and yields of harvested silage and grazed temporary grasslands, expressed in ton dry matter (DM) per hectare (ha) used when comparing the profitability of various combinations of cattle production systems, grass silages (timothy, meadow fescue, and timothy-meadow fescue are traditional grasses (TR) and tall fescue, festulolium, and reed canary grass are alternative wet-and-drought-resistant grasses (WD)), and weather scenarios (historically normal weather, Reference (Ref), wet weather with two weeks delay of harvest (Wet) and dry weather with 216 mm lower precipitation than normal (Dry))

Item	Gsk	Gns	Nn
Full name	Forest districts in southern Sweden	Plain districts in southern Sweden	Lower parts of northern Sweden
Field configuration	Scattered fields, irregular shapes	Large fields, rectangular shape	Scattered fields, irregular shapes
Farm size[a], ha	150	500	150
Harvested silage yield, ton DM/ha			
Timothy[b]			
Ref	8.7	10.5	6.8
Wet	9.8	11.9	7.6
Dry	5.5	6.6	4.3
Meadow fescue[b]			
Ref	9.3	9.9	6.0
Wet	10.4	11.0	6.7
Dry	5.9	6.2	3.7
Timothy-meadow fescue[b]			
Ref	9.0	10.2	6.4
Wet	9.0	10.2	6.4
Dry	5.7	6.4	4.0
Tall fescue[b]			
Ref	9.5	11.1	6.7
Wet	10.7	12.4	7.5
Dry	8.0	8.3	5.7
Festulolium[b]			
Ref	10.2	11.9	7.0
Wet	10.2	11.9	7.0
Dry	7.7	8.9	5.2
Reed canary grass[c]			
Ref	8.6	8.6	8.2
Wet	8.6	8.6	8.2
Dry	5.6	5.6	5.4
Grazed herbage yield[d], ton DM/ha			
Ref	4.5	5.1	3.7
Wet	4.5	5.1	3.7
Dry	2.9	3.2	2.3

Footnote: When calculating the need of additional area of semi-natural pastures to beef cows in the Dry scenario, grazing low-opportunity land was supposed.

References

[a]Berlin-Thorell K., 2020. Advisor, Rådgivarna i Sjuhärad, Länghem, Sweden. Personal communication.

[b]Halling M., Sandström B., Hallin O. & Larsson S., 2023. Vallväxter till slåtter och bete samt grönfoderväxter. Sortval för södra, mellersta och norra Sverige 2020/2021 / Forage species for cutting, grazing and green fodder. Varieties for south, central and northern Sweden 2020/2021.

[c]Palmborg C., 2016. Reed canary grass – In depth studies of varieties Department of agricultural research for northern Sweden. Swedish University of Agricultural Sciences, Umeå (1). https://pub.epsilon.slu.se/13401/7/palmborg_c_160524.pdf

[d]Agriwise, 2023. Agriwise - Smart kalkylering. https://www.agriwise.se/web (accessed 18 January 2024).

Table S3. Method and data used for calculating revenues and variable costs when comparing the profitability of various combinations of cattle production systems, grass silages, weather scenarios, and geographical regions in Sweden; calculated as average price 2019 – 2023 for milk, calf, carcass, calf purchase, concentrate and labour. Other prices are collected from sources with prices originating from different years. Those sums are index calculated to the same average price level as above by using series of indexes for different means of production[a]; 11.53 SEK = 1 Euro; ha = hectare

	Description
Revenues	
Milk	Milk yield[b] 305 d (delivered yield 0.94) × average price paid at Arla corrected for content of fat and protein[c]
Calf	Calf[d] × price paid for calf[e]
Carcass	Carcass weight × average price paid at abattoir for cows and bulls at different classifications[e,f]
Animal premium	Days >1 year/365 × 91 Euro[g]
Support for milk production in Nn	Delivered milk yield × 0.1 Euro/kg milk in Nn[g]
Support to hoof care	No. of dairy cow × 26 Euro/cow[g]
Manure	Tons of manure from animal[h] × content and price for N, P, K/ton[f]
Variable costs	
Calf	Price paid for calf[d]
Replacement	Price paid per replacement heifer × % replaced cows[d]
Grass silage	Kg DM × cost of production for silage in different regions and weather conditions calculated as described in Table A2b, 0.07-0.29 Euro/kg DM depending on grass silages
Concentrate (cow compound feed, grain, mineral supplement)	Kg concentrate for dairy × price for cow-concentrate 0.35 Euro/kg[i]. Kg grain × price for barley/wheat 0.16 Euro/kg (selling)[d]. Kg mineral × price for minerals 0.646 Euro/kg[i]
Grazed herbage	Kg DM,[d,j] × cost for grazed herbage in different regions calculated as described in Table A2b, -0.01-0.25 Euro/kg DM grass on arable land and -0.14 - (-)0.37 Euro/kg DM on semi-natural pasture
Bedding	Kg of wood shaving[d] × 0.09 Euro/kg[d]
Various cost	Veterinary, medicine, and assurance[d]
Maintenance inventories	Cost for maintenance inventories [d]
Maintenance building inventories	Cost for maintenance building inventories [d]
Building maintenance cost	Building cost[k] x 0.25% yearly maintenance[d]
Labour	Labour, hours (dairy cow[d]; beef cow[l]; beef bull[m,n] × wage[d]
Building inventories depreciation and interest	Cost of building inventories[k] – investment support (30% of building cost but not more than 0.21 MEuro/farm). Annuity at depreciation 15 years for equipment, 4% interest rate[d]
Building depreciation and interest	Building cost[f] – investment support (30% of building cost but not more than 0.21 MEuro/farm). Annuity at depreciation 30 years for building structure, 4% interest rate[d]
Interest working capital	Rearing time/year × variable costs exclusive calf purchase, replacement heifer × 0.55 (only for beef bull) × 4% interest rate[d]
Interest animal capital	Rearing time/year × (replacement heifer + carcass income from cow)/2 or calf purchase × 4% interest rate[d]

References

[a]Swedish Board of Agriculture, 2023. Agricultural statistics. https://jordbruksverket.se/om-jordbruksverket/jordbruksverkets-officiella-statistik/jordbruksstatistisk-sammanstallning (accessed 18 January 2024).

[b]Sousa, D. O., Murphy, M., Hatfield, R., & Nadeau, E., 2021. Effects of harvest date and grass species on silage cell wall components and lactation performance of dairy cows. *Journal of Dairy Science*, *104*(5), 5391–5404. https://doi.org/10.3168/jds.2020-19362

[c]Arla, 2023. Arlapris. https://www.arla.se/om-arla/agare/arlapris/ (accessed 18 January 2024).

[d]Agriwise, 2023. Agriwise - Smart kalkylering. https://www.agriwise.se/web (accessed 18 January 2024).

[e]HKScan Agri, 2023. HKScan Agri notering. https://www.hkscanagri.se/notering/ (accessed 18 January 2024).

[f]Swedish Board of Agriculture, 2023. Priser och marknadsinformation för livsmedel. https://jordbruksverket.se/mat-och-drycker/handel-och-marknad/priser-och-marknadsinformation-for-livsmedel (accessed 18 January 2024).

[g]Swedish Board of Agriculture, 2023. Stöd till lantbrukare och verksamma på landsbygden. https://jordbruksverket.se/stod (accessed 18 January 2024).

[h]Swedish Board of Agriculture, 2024. Rekommendationer för gödsling och kalkning 2024. https://webbutiken.jordbruksverket.se/sv/artiklar/jo219.html (accessed 5 May 2024).

[i]Öhman K., 2024. Lantmännen. Personal communication.

[j]Jardstedt M., Nadeau E., Olaf Nielsen M., Nørgaard P., and Hessle A., 2019. The effect of feeding roughages of varying digestibility prepartum on energy status and metabolic profiles in beef cows around parturition. *Animals* (3).

[k]Hedlund S. 2023. Hushållningssällskapet Västra. Personal communication.

[l]Holmström K., Andersson H., Kumm K.-I., and Hessle A. 2023. Labour in suckler cow herds – a study on enterprises in southern Sweden. Acta Agriculturae Scandinavica, section A-Animal science. https://doi.org/10.1080/09064702.2023.2245400

[m]Bostad E., Swensson C., and Pinzke S., 2011. Labour input in specialist beef bull production in Sweden. Agricultural Engineering International: *CIGR J.* http://cigrjournal.org/index.php/Ejounral/article/view/1920

[n]Nelson B.-O., 2002. Kalkylmodell för nötköttsproduktion. Skogs- och lantarbetsgivareförbundets analysgrupp.

Table S4. Method and data used for calculating revenues and costs in silage and pasture production when comparing the profitability of various combinations of cattle production systems, grass silages, weather scenarios, and geographical regions in Sweden; calculated as average price 2019 – 2023 for seed, fertilizer, machinery pasture, fence, labour, and opportunity cost. Other costs are collected from sources with prices originating from different years. Those sums are index calculated to the same average price level as above by using series of indexes for different means of production[a] (Swedish Board of Agriculture, 2023a); 11.53 SEK = 1 Euro; ha = hectare

	Description
Revenues	
Single farm payment	No. of ha arable land and semi-natural pastures × 144 Euro/ha[a]
Agri-environmental payment	No. of ha semi-natural pastures × 161 Euro/ha for ordinary semi-natural pasture (50% of area) and 343 Euro/ha for especially valuable semi-natural pasture[a]
Support to LFA	No. of ha arable land and semi-natural pastures × depending on livestock density 121-182 Euro/ha (Gsk) or 252-338 Euro/ha (Nn)[a]
Costs	
Seed	Seed sown, kg × average price[b], divided with three years (five years for meadow fescue-timothy and ten years for reed canary grass)
Fertilizer	N, P and K, kg adjusted to harvest level[c] × average price[b]
Machinery, silage	Kg dry matter (DM) × machinery cost per kg DM silage in different regions, 0.071-0.291 Euro/kg DM[d]
Plastic film	Kg DM × cost for plastic film per kg DM[d]
Machinery, pasture	Fuel and maintenance for tractor and machinery[b]
Fence, maintenance	Cost of fence × 4% yearly maintenance cost[b]
Labour	Working hours for fencing and maintenance × wage[b]
Opportunity cost	No. of ha × 189-655 Euro/ha[a] for agricultural land and semi-natural pasture in different regions of Sweden
Fence, capital cost	Cost for fence × annuity for 12 years and 4% interest rate[b]
Interest working capital	Variable costs × 0.5 × 4% interest rate[b]

References

[a]Swedish Board of Agriculture, 2023. Stöd till lantbrukare och verksamma på landsbygden. https://jordbruksverket.se/stod (accessed 18 January 2024).

[b]Agriwise, 2023. Agriwise - Smart kalkylering. https://www.agriwise.se/web (accessed 18 January 2024).

[c]Swedish Board of Agriculture, 2024. Rekommendationer för gödsling och kalkning 2024. https://webbutiken.jordbruksverket.se/sv/artiklar/jo219.html (accessed 5 May 2024).

[d]Neuman L., 2024. Spirina consult, Timmele, Sweden. Personal communication.

Table S5a. Contribution margin (= contribution to common cost, risk, and profit) in a herd of 100 dairy cows located in forest districts of Götaland (Gsk), where cows are fed grass silages of either traditional (TR) timothy or wet-and-drought-resistant (WD) tall fescue under three different weather scenarios; historically normal weather, Reference (Ref), wet weather with two weeks delayed grass cut compared to Reference (Wet); and dry weather with 216 mm lower precipitation and hence 26-37% lower herbage yield than in Ref (Dry), Euro per cow and year

	Ref		Wet		Dry	
	TR	WD	TR	WD	TR	WD
Revenues						
Milk	3 821	3 386	3 533	3 461	3 821	3 386
Calf, heifer	4	4	4	4	4	4
Calf, bull	7	7	7	7	7	7
Culled cow	423	423	423	423	423	423
Animal premium	91	91	91	91	91	91
Support for hoof care	26	26	26	26	26	26
Support for milk production in Nn	0	0	0	0	0	0
Manure	288	248	248	242	288	248
Sum revenues	4 658	4 184	4 331	4 253	4 658	4 184
Variable costs						
Replacement heifer	422	422	422	422	422	422
Milk replacer	138	138	138	138	138	138
Silage	508	419	395	425	674	441
Grazed herbage	8	8	8	8	12	12
Concentrate (cow compound feed)	1 561	1 361	1 282	1 392	1 666	1 453
Bedding	35	35	35	35	35	35
Various cost (veterinary, medicine, assurance)	347	347	347	347	347	347
Labour	469	469	469	469	469	469
Building and inventories, maintenance	49	49	49	49	49	49
Inventories depreciation and interest	228	228	228	228	228	228
Building, depreciation and interest	227	227	227	227	227	227
Interest working capital	62	57	54	57	68	59
Interest animal capital	41	41	41	41	41	41
Sum variable cost	4 093	3 799	3 694	3 837	4 375	3 920
Contribution margin	566	385	637	417	283	263

Table S5b. Contribution margin (=contribution to common cost, risk, and profit) in a herd of 100 dairy cows located in plain districts in northern Götaland (Gns), where cows are fed grass silages of either traditional (TR) timothy or wet-and-drought-resistant (WD) tall fescue under three different weather scenarios; historically normal weather, Reference (Ref), wet weather with two weeks delayed grass cut compared to Reference (Wet); and dry weather with 216 mm lower precipitation and hence 26-37% lower herbage yield than in Ref (Dry), Euro per cow and year

	Ref		Wet		Dry	
	TR	WD	TR	WD	TR	WD
Revenues						
Milk	3 821	3 386	3 533	3 461	3 821	3 386
Calf, heifer	4	4	4	4	4	4
Calf, bull	7	7	7	7	7	7
Culled cow	423	423	423	423	423	423
Animal premium	91	91	91	91	91	91
Support for hoof care	26	26	26	26	26	26
Support for milk production in Nn	0	0	0	0	0	0
Manure	288	248	248	242	288	248
Sum revenues	4 658	4184	4 331	4 253	4 658	4 184
Variable costs						
Replacement heifer	422	422	422	422	422	422
Milk replacer	138	138	138	138	138	138
Silage	677	539	517	542	941	668
Grazed herbage	18	18	18	18	29	29
Concentrate (cow compound feed)	1 561	1 361	1 282	1 392	1 666	1 453
Bedding	35	35	35	35	35	35
Various cost (veterinary, medicine, assurance)	347	347	347	347	347	347
Labour	469	469	469	469	469	469
Building and inventories, maintenance	49	49	49	49	49	49
Inventories depreciation and interest	228	228	228	228	228	228
Building, depreciation and interest	227	227	227	227	227	227
Interest working capital	66	59	57	60	73	64
Interest animal capital	41	41	41	41	41	41
Sum variable cost	4 276	3 932	3 829	3 967	4 665	4 169
Contribution margin	382	252	502	286	-7	15

Table S5c. Contribution margin (= contribution to common cost, risk, and profit) in a herd of 100 dairy cows located in lower parts of Norrland (Nn), where cows are fed grass silages of either traditional (TR) timothy or wet-and-drought-resistant (WD) tall fescue under three different weather scenarios; historically normal weather, Reference (Ref), wet weather with two weeks delayed grass cut compared to Reference (Wet); and dry weather with 216 mm lower precipitation and hence 26-37% lower herbage yield than in Ref (Dry), Euro per cow and year

	Ref		Wet		Dry	
	TR	WD	TR	WD	TR	WD
Revenues						
Milk	3 821	3 386	3 533	3 461	3 821	3 386
Calf, heifer	4	4	4	4	4	4
Calf, bull	7	7	7	7	7	7
Culled cow	423	423	423	423	423	423
Animal premium	91	91	91	91	91	91
Support for hoof care	26	26	26	26	26	26
Support for milk production in Nn	1 036	897	947	897	1 036	897
Manure	288	248	248	242	288	248
Sum revenues	5 694	5 081	5 278	5 150	5 694	5 081
Variable costs						
Replacement heifer	422	422	422	422	422	422
Milk replacer	138	138	138	138	138	138
Silage	424	337	323	355	511	353
Grazed herbage	-1	-1	-1	-1	-1	-1
Concentrate (cow compound feed)	1 561	1 361	1 282	1 392	1 666	1 453
Bedding	35	35	35	35	35	35
Various cost (veterinary, medicine, assurance)	347	347	347	347	347	347
Labour	469	469	469	469	469	469
Building and inventories, maintenance	49	49	49	49	49	49
Inventories depreciation and interest	228	228	228	228	228	228
Building, depreciation and interest	227	227	227	227	227	227
Interest working capital	60	55	53	56	64	57
Interest animal capital	41	41	41	41	41	41
Sum variable cost	3 999	3 706	3 612	3 756	4 195	3 816
Contribution margin	1 696	1 375	1 666	1 394	1 499	1 264

Table S6a. Contribution margin (= contribution to common cost, risk, and profit) in a herd of 50 reared beef breed bulls per year located in forest districts of Götaland (Gsk), where bulls are fed grass silages of either traditional (TR) meadow fescue or wet-and-drought-resistant (WD) tall fescue under three different weather scenarios; historically normal weather, Reference (Ref), wet weather with two weeks delayed grass cut compared to Reference (Wet); and dry weather with 216 mm lower precipitation and hence 26-37% lower herbage yield than in Ref (Dry), Euro per reared bull

	Ref		Wet		Dry	
	TR	WD	TR	WD	TR	WD
Revenues						
Carcass	1 636	1 598	1 636	1 598	1 636	1 598
Animal premium	65	61	70	65	65	61
Manure	53	51	53	51	53	51
Sum revenues	1 754	1 710	1 760	1 714	1 754	1 710
Variable costs						
Calf	760	760	760	760	760	760
Silage	286	301	287	292	395	319
Concentrate (grain)	117	111	128	118	146	138
Mineral	8	8	8	8	8	8
Bedding	63	63	69	68	63	63
Various cost (veterinary, medicine, assurance)	31	31	31	31	31	31
Labour	104	98	113	105	104	98
Building and inventories, maintenance	15	14	17	16	15	14
Inventories depreciation and interest	61	61	61	61	61	61
Building, depreciation and interest	186	186	186	186	186	186
Interest working capital	10	9	11	10	12	10
Interest animal capital	22	21	24	22	22	21
Sum variable cost	1 663	1 663	1 695	1 676	1 804	1 710
Contribution margin	91	47	65	38	-50	0

Table S6b. Contribution margin (= contribution to common cost, risk, and profit) in a herd of 50 reared beef breed bulls per year located in plain districts in northern Götaland (Gns), where bulls are fed grass silages of either traditional (TR) meadow fescue or wet-and-drought-resistant (WD) tall fescue under three different weather scenarios; historically normal weather, Reference (Ref), wet weather with two weeks delayed grass cut compared to Reference (Wet); and dry weather with 216 mm lower precipitation and hence 26-37% lower herbage yield than in Ref (Dry), Euro per reared bull

	Ref		Wet		Dry	
	TR	WD	TR	WD	TR	WD
Revenues						
Carcass	1 636	1 598	1 636	1 598	1 636	1 598
Animal premium	65	61	70	65	65	61
Manure	53	51	53	51	53	51
Sum revenues	1 754	1 710	1 760	1 714	1 754	1 710
Variable costs						
Calf	760	760	760	760	760	760
Silage	377	368	368	355	534	456
Concentrate (grain)	117	111	128	118	146	138
Mineral	8	8	8	8	8	8
Bedding	63	63	69	68	63	63
Various cost (veterinary, medicine, assurance)	31	31	31	31	31	31
Labour	104	98	113	105	104	98
Building and inventories, maintenance	15	14	17	16	15	14
Inventories depreciation and interest	61	61	61	61	61	61
Building, depreciation and interest	186	186	186	186	186	186
Interest working capital	11	10	13	11	14	12
Interest animal capital	22	21	24	22	22	21
Sum variable cost	1 755	1 731	1 777	1 740	1 944	1 848
Contribution margin	-1	-21	-17	-26	-190	-139

Table S6c. Contribution margin (= contribution to common cost, risk, and profit) in a herd of 50 reared beef breed bulls per year located in lower parts of Norrland (Nn), where bulls are fed grass silages of either traditional (TR) meadow fescue or wet-and-drought-resistant (WD) tall fescue under three different weather scenarios; historically normal weather, Reference (Ref), wet weather with two weeks delayed grass cut compared to Reference (Wet); and dry weather with 216 mm lower precipitation and hence 26-37% lower herbage yield than in Ref (Dry), Euro per reared bull

	Ref		Wet		Dry	
	TR	WD	TR	WD	TR	WD
Revenues						
Carcass	1 636	1 598	1 636	1 598	1 636	1 598
Animal premium	65	61	70	65	65	61
Manure	53	51	53	51	53	51
Sum revenues	1 754	1 710	1 760	1 714	1 754	1 710
Variable costs						
Calf	760	760	760	760	760	760
Silage	257	260	257	260	340	277
Concentrate (grain)	117	111	128	118	146	138
Mineral	8	8	8	8	8	8
Bedding	63	63	69	68	63	63
Various cost (veterinary, medicine, assurance)	31	31	31	31	31	31
Labour	104	98	113	105	104	98
Building and inventories, maintenance	15	14	17	16	15	14
Inventories depreciation and interest	61	61	61	61	61	61
Building, depreciation and interest	186	186	186	186	186	186
Interest working capital	9	9	11	10	11	9
Interest animal capital	22	21	24	22	22	21
Sum variable cost	1 634	1 622	1 664	1 644	1 747	1 667
Contribution margin	120	88	96	70	7	43

Table S7a. Contribution margin (= contribution to common cost, risk, and profit) in a herd of 50 beef cows located in forest districts of Götaland (Gsk) lower parts of, where cows are fed grass silages of either traditional (TR) meadow fescue-timothy, wet-and-drought-resistant (WD-f) festulolium, or wet-and-drought-resistant (WD-r) reed canary grass under three different weather scenarios; historically normal weather, Reference (Ref), wet weather with two weeks delayed grass cut compared to Reference (Wet); and dry weather with 216 mm lower precipitation and hence 26-37% lower herbage yield than in Ref (Dry), Euro per cow and year

	Ref			Wet			Dry		
	TR	WD-f	WD-r	TR	WD-f	WD-r	TR	WD-f	WD-r
Revenues									
Calf, heifer	241	241	241	241	241	241	241	241	241
Calf, bull	324	324	324	324	324	324	324	324	324
Culled cow	251	251	251	251	251	251	251	251	251
Animal premium	91	91	91	91	91	91	91	91	91
Manure	51	51	51	51	51	51	51	51	51
Sum revenues	957	957	957	957	957	957	957	957	957
Variable costs									
Replacement heifer	236	236	236	236	236	236	236	236	236
Silage	311	312	181	311	312	181	415	563	206
Grazed herbage	-566	-542	-618	-566	-542	-618	-566	-542	-618
Mineral	17	17	17	17	17	17	17	17	17
Bedding	62	62	62	62	62	62	62	62	62
Various cost (vet., medicine, assurance)	90	90	90	90	90	90	90	90	90
Labour	384	384	384	384	384	384	384	384	384
Building and inventories, maintenance	9	9	9	9	9	9	9	9	9
Inventories depreciation and interest	135	135	135	135	135	135	135	135	135
Building, depreciation and interest	250	250	250	250	250	250	250	250	250
Interest working capital	7	7	2	7	7	2	8	12	2
Interest animal capital	49	49	49	49	49	49	49	49	49
Sum variable cost	983	1 009	796	983	1 009	796	1 089	1 265	822
Contribution margin	-26	-52	161	-26	-52	161	-132	-307	136

Table S7b. Contribution margin (= contribution to common cost, risk, and profit) in a herd of 50 beef cows located in plain districts in northern Götaland (Gns) lower parts of, where cows are fed grass silages of either traditional (TR) meadow fescue-timothy, wet-and-drought-resistant (WD-f) festulolium, or wet-and-drought-resistant (WD-r) reed canary grass under three different weather scenarios; historically normal weather, Reference (Ref), wet weather with two weeks delayed grass cut compared to Reference (Wet); and dry weather with 216 mm lower precipitation and hence 26-37% lower herbage yield than in Ref (Dry), Euro per cow and year

	Ref			Wet			Dry		
	TR	WD-f	WD-r	TR	WD-f	WD-r	TR	WD-f	WD-r
Revenues									
Calf, heifer	241	241	241	241	241	241	241	241	241
Calf, bull	324	324	324	324	324	324	324	324	324
Culled cow	251	251	251	251	251	251	251	251	251
Animal premium	91	91	91	91	91	91	91	91	91
Manure	51	51	51	51	51	51	51	51	51
Sum revenues	957	957	957	957	957	957	957	957	957
Variable costs									
Replacement heifer	236	236	236	236	236	236	236	236	236
Silage	431	441		431	441		610	543	402
Grazed herbage	-307	-294	336	-307	-294	-336	-307	-294	-336
Mineral	17	17	17	17	17	17	17	17	17
Bedding	62	62	62	62	62	62	62	62	62
Various cost (vet., medicine, assurance)	90	90	90	90	90	90	90	90	90
Labour	384	384	384	384	384	384	384	384	384
Building and inventories, maintenance	9	9	9	9	9	9	9	9	9
Inventories depreciation and interest	135	135	135	135	135	135	135	135	135
Building, depreciation and interest	250	250	250	250	250	250	250	250	250
Interest working capital	14	14	10	14	14	10	17	16	10
Interest animal capital	49	49	49	49	49	49	49	49	49
Sum variable cost	1 369	1 392	1 200	1 369	1 392	1 200	1 551	1 496	1 309
Contribution margin	-412	-435	-243	-412	-435	-243	-594	-539	-351

Table S7c. Contribution margin (= contribution to common cost, risk, and profit) in a herd of 50 beef cows located in lower parts of Norrland (Nn), where cows are fed grass silages of either traditional (TR) meadow fescue-timothy, wet-and-drought-resistant (WD-f) festulolium, or wet-and-drought-resistant (WD-r) reed canary grass under three different weather scenarios; historically normal weather, Reference (Ref), wet weather with two weeks delayed grass cut compared to Reference (Wet); and dry weather with 216 mm lower precipitation and hence 26-37% lower herbage yield than in Ref (Dry), Euro per cow and year

	Ref			Wet			Dry		
	TR	WD-f	WD-r	TR	WD-f	WD-r	TR	WD-f	WD-r
Revenues									
Calf, heifer	241	241	241	241	241	241	241	241	241
Calf, bull	324	324	324	324	324	324	324	324	324
Culled cow	251	251	251	251	251	251	251	251	251
Animal premium	91	91	91	91	91	91	91	91	91
Manure	51	51	51	51	51	51	51	51	51
Sum revenues	957	957	957	957	957	957	957	957	957
Variable costs									
Replacement heifer	236	236	236	236	236	236	236	236	236
Silage	271	299	140	271	299	140	325	334	148
Grazed herbage	-667	-632	-743	-667	-632	-743	-667	-632	-743
Mineral	17	17	17	17	17	17	17	17	17
Bedding	62	62	62	62	62	62	62	62	62
Various cost (vet., medicine, assurance)	90	90	90	90	90	90	90	90	90
Labour	384	384	384	384	384	384	384	384	384
Building and inventories, maintenance	9	9	9	9	9	9	9	9	9
Inventories depreciation and interest	135	135	135	135	135	135	135	135	135
Building, depreciation and interest	250	250	250	250	250	250	250	250	250
Interest working capital	5	5	-1	5	5	-1	4	5	2
Interest animal capital	49	49	49	49	49	49	49	49	49
Sum variable cost	841	903	628	841	903	628	894	938	640
Contribution margin	117	54	329	117	54	329	63	19	317

Table S8a. Contribution margin (= contribution to common cost, risk, and profit) in calculation with varying proportions of years with different weathers; historically normal weather, Reference (Ref), wet weather with delayed harvest (Wet), and dry weather with lower grass yield (Dry), when feeding grass silages of either traditional (TR) timothy or wet-and-drought resistant (WD) tall fescue in a herd with 100 dairy cows, located in forest districts (Gsk), plain districts (Gns), or northern districts (Nn) of Sweden, Euro per cow and year

Proportion of weather			*Gsk*		*Gns*		*Nn*	
Ref	Wet	Dry	TR	WD	TR	WD	TR	WD
1.00	0.00	0.00	566	385	382	252	1696	1375
0.50	0.50	0.00	601	401	442	269	1681	1384
0.50	0.00	0.50	424	324	188	133	1597	278
0.25	0.75	0.00	619	409	472	277	1674	285
0.25	0.00	0.75	354	294	90	74	1548	256
0.00	1.00	0.00	637	417	502	286	1666	273
0.00	0.75	0.25	548	378	375	218	1625	263
0.00	0.25	0.75	372	302	120	82	1541	244
0.00	0.00	1.00	283	263	-7	15	1499	235

Table S8b. Contribution margin (= contribution to common cost, risk, and profit) in calculation with varying proportions of years with different weathers; historically normal weather, Reference (Ref), wet weather with delayed harvest (Wet), and dry weather with lower grass yield (Dry), when feeding grass silages of either traditional (TR) meadow fescue or wet-and-drought resistant (WD) tall fescue, in a herd with 50 slaughtered beef breed bulls per year located in forest districts (Gsk), plain districts (Gns), or northern districts (Nn) of Sweden, Euro per reared bull

Proportion of weather			*Gsk*		*Gns*		*Nn*	
Ref	Wet	Dry	TR	WD	TR	WD	TR	WD
1.00	0.00	0.00	91	47	-1	-21	120	88
0.50	0.50	0.00	78	42	-9	-24	108	79
0.50	0.00	0.50	21	23	-96	-80	63	65
0.25	0.75	0.00	72	40	-13	-25	102	74
0.25	0.00	0.75	-15	12	-143	-109	35	54
0.00	1.00	0.00	65	38	-17	-26	96	70
0.00	0.75	0.25	36	28	-60	-54	73	63
0.00	0.25	0.75	-21	10	-147	-110	29	49
0.00	0.00	1.00	-50	0	-190	-139	7	43

Table S8c. Contribution margin (= contribution to common cost, risk, and profit) in calculation with varying proportions of years with different weathers; historically normal weather, Reference (Ref), wet weather with delayed harvest (Wet), and dry weather with lower grass yield (Dry), when feeding grass silages of either traditional (TR) meadow fescue-timothy, wet-and-drought resistant festulolium (WD-f), or reed canary grass (WD-r) in a herd with 50 beef suckler cows located in forest districts (Gsk), plain districts (Gns), or northern districts (Nn) of Sweden, Euro per cow and year

Proportion of weather			*Gsk*			*Gns*			*Nn*		
Ref	Wet	Dry	TR	WD-f	WD-r	TR	WD-f	WD-r	TR	WD-f	WD-r
1.00	0.00	0.00	-26	-52	161	-412	-435	-243	117	54	329
0.50	0.50	0.00	-26	-52	161	-412	-435	-243	117	54	329
0.50	0.00	0.50	-79	-95	148	-503	-487	-299	90	37	316
0.25	0.75	0.00	-26	-59	161	-412	-435	-247	117	54	314
0.25	0.00	0.75	-105	-114	142	-549	-513	-325	76	28	317
0.00	1.00	0.00	-26	-59	161	-412	-435	-247	117	54	314
0.00	0.75	0.25	-52	-77	154	-457	-461	-273	103	46	315
0.00	0.25	0.75	-105	-114	142	-549	-513	-325	76	28	317
0.00	0.00	1.00	-132	-132	136	-594	-539	-351	63	19	317

Table S9. Cost for silage production in a basic calculation and a sensitivity analysis with rent cost instead of opportunity cost for arable land in herds with 100 dairy cows, 50 beef breed bulls, or 50 beef suckler cows, fed grass silages of either traditional (TR) grasses (timothy for dairy cow, meadow fescue for bull, meadow fescue-timothy for beef cow) or wet-and-drought resistant (WD) grasses (tall fescue for dairy cow and bull, festulolium (f) or reed canary grass (r) for beef cow) in forest districts (Gsk), plain districts (Gns), and northern districts (Nn) of Sweden in three different weather scenarios; historically normal weather, Reference (Ref), wet weather with delayed harvest (Wet), and dry weather with lower grass yield (Dry). Euro per kg dry matter

	Dairy cow		Beef breed bull		Beef cow		
	TR	WD	TR	WD	TR	WD-f	WD-r
Gsk							
Basic calculation							
Ref	0.150	0.151	0.156	0.159	0.134	0.125	0.109
Wet	0.142	0.141	0.148	0.148	0.134	0.125	0.109
Dry	0.199	0.159	0.216	0.168	0.179	0.225	0.131
Rent cost							
Ref	0.122	0.125	0.129	0.133	0.106	0.101	0.081
Wet	0.117	0.117	0.124	0.125	0.106	0.101	0.081
Dry	0.154	0.128	0.173	0.137	0.135	0.177	0.084
Gns							
Basic calculation							
Ref	0.200	0.194	0.206	0.194	0.186	0.176	0.178
Wet	0.186	0.180	0.189	0.180	0.186	0.176	0.178
Dry	0.278	0.241	0.291	0.241	0.263	0.217	0.255
Rent cost							
Ref	0.140	0.136	0.141	0.136	0.123	0.123	0.104
Wet	0.132	0.128	0.131	0.128	0.123	0.123	0.104
Dry	0.182	0.164	0.189	0.164	0.163	0.145	0.136
Nn							
Basic calculation							
Ref	0.125	0.121	0.141	0.137	0.098	0.100	0.071
Wet	0.116	0.118	0.132	0.132	0.098	0.100	0.071
Dry	0.151	0.127	0.186	0.146	0.117	0.112	0.079
Rent cost							
Ref	0.102	0.098	0.114	0.114	0.073	0.077	0.052
Wet	0.095	0.097	0.109	0.111	0.073	0.077	0.052
Dry	0.114	0.099	0.143	0.118	0.078	0.081	0.048

Table S10. Cost for grazed herbage on temporary grasslands in a basic calculation and a sensitivity analysis with rent cost instead of opportunity cost for land herds with 100 dairy cows in forest districts (Gsk), plain districts (Gns), and northern districts (Nn) of Sweden in three different weather scenarios; historically normal weather, Reference (Ref), wet weather with delayed harvest (Wet), and dry weather with lower grass yield (Dry). Euro per kg dry matter

	Basic calculation			Rent cost		
	Ref	Wet	Dry	Ref	Wet	Dry
Gsk	0.064	0.064	0.104	0.020	0.020	0.034
Gns	0.156	0.156	0.249	0.056	0.056	0.091
Nn	-0.009	-0.009	-0.012	-0.043	-0.043	-0.067

Table S11a. Contribution margin (= contribution to common cost, risk, and profit) in a basic calculation and a sensitivity analysis with rent cost instead of opportunity cost for arable land, in a herd with 100 dairy cows fed grass silages of either traditional (TR) timothy or wet-and-drought resistant (WD) tall fescue located in forest districts (Gsk), plain districts (Gns), or northern districts (Nn) of Sweden in three different weather scenarios; historically normal weather, Reference (Ref), wet weather with delayed harvest (Wet), and dry weather with lower grass yield (Dry), Euro per cow and year

	Ref		Wet		Dry	
	TR	WD	TR	WD	TR	WD
Gsk						
Basic calculation	566	385	637	417	283	263
Rent cost	669	464	714	494	447	360
Gns						
Basic calculation	382	252	502	286	-7	15
Rent cost	603	427	667	456	344	252
Nn						
Basic calculation	1 696	1 375	1 666	1 394	1 499	1 264
Rent cost	1 780	1 445	1 730	1 463	1 634	1 350

Table S11b. Contribution margin (= contribution to common cost, risk, and profit) in a basic calculation and a sensitivity analysis with rent cost instead of opportunity cost for arable land in a herd with 50 slaughtered beef breed bulls per year fed grass silages of either traditional (TR) meadow fescue or wet-and-drought resistant (WD) tall fescue located in forest districts (Gsk), plain districts (Gns), and northern districts (Nn) of Sweden in three different weather scenarios; historically normal weather, Reference (Ref), wet weather with delayed harvest (Wet), and dry weather with lower grass yield (Dry), Euro per reared bull

	Ref		Wet		Dry	
	TR	WD	TR	WD	TR	WD
Gsk						
Basic calculation	91	47	65	38	-50	0
Rent cost	141	97	112	85	29	60
Gns						
Basic calculation	-1	-21	-17	-26	-190	-139
Rent cost	119	90	97	77	1	9
Nn						
Basic calculation	120	88	96	70	7	43
Rent cost	169	133	143	112	85	96

Table S11c. Contribution margin (= contribution to common cost, risk, and profit) in a basic calculation and a sensitivity analysis with rent cost instead of opportunity cost for arable land in a herd with 50 beef suckler cows fed grass silages of either traditional (TR) meadow fescue-timothy, wet-and-drought resistant festulolium(WD-f), or reed canary grass (WD-r) in forest districts (Gsk), plain districts (Gns), and northern districts (Nn) of Sweden in three different weather scenarios; historically normal weather, Reference (Ref), wet weather with delayed harvest (Wet), and dry weather with lower grass yield (Dry), Euro per cow and year

	Ref			Wet			Dry		
	TR	WD-f	WD-r	TR	WD-f	WD-r	TR	WD-f	WD-r
Gsk									
Basic calculation	-26	-52	161	-26	-52	161	-132	-307	136
Rent cost	39	4	209	39	4	209	-28	-183	210
Gns									
Basic calculation	-412	-435	-243	-412	-435	-243	-594	-539	-351
Rent cost	-263	-298	-122	-263	-298	-122	-359	-356	-161
Nn									
Basic calulation	117	54	329	117	54	329	63	19	317
Rent cost	187	123	355	187	123	355	175	111	377
